# Influence of eating while watching TV on food preference and overweight/obesity among adolescents in China: a longitudinal study

**DOI:** 10.3389/fpubh.2024.1423383

**Published:** 2024-09-17

**Authors:** Jinli Xian, Tingwei Ren, Ming Kuang

**Affiliations:** Department of Clinical Nutrition, MianYang Central Hospital, Mianyang, Sichuan, China

**Keywords:** television, meals, snacks, food preference, overweight, adolescents, China

## Abstract

**Background:**

Eating while watching TV was found associated with unhealthy food preferences and obesity in adolescents in foreign studies, which is not clear in China. The study aims to explore the influence of eating while watching TV on food preferences and overweight/obesity in Chinese adolescents.

**Methods:**

Data from 1768 adolescents (aged 12–17 years) in the 2006, 2009, 2011, and 2015 China Health and Nutrition Survey (CHNS) was analyzed. The height and weight were measured. Mixed effect models were used to identify the associations between eating while watching TV and adolescents’ food preferences and overweight/obesity.

**Results:**

Adolescents eating while watching TV ≥1 time/week were more likely to prefer fast food, salty snacks and soft drinks than those eating while watching TV <1 time/week. Adolescents eating meals while watching TV ≥1 time/week were less likely to prefer vegetables than those eating meals while watching TV <1 time/week. In addition, adolescents eating snacks while watching TV ≥1 time/week were more likely to be overweight/obesity than those eating meals while watching TV <1 time/week (odds ratio [OR] = 7.16; 95% confidence interval [CI] 1.39–36.93).

**Conclusion:**

Eating snacks while watching TV was positively associated with adolescents’ unhealthy food preferences and overweight/obesity. Eating meals while watching TV was associated with adolescents’ unhealthy food preferences. Implementing web-based Community-based participatory research (CBPR) about reducing eating while watching TV may be a practical strategy to develop healthy food preferences and prevent overweight/obesity in Chinese adolescents.

## 1 Introduction

Adolescence is a critical period for children’s intelligence and body growth and development. However, the prevalence of overweight and obesity among children and adolescents in China has been increasing for decades (1). Unhealthy dietary habits were known high-risk factors for children’s obesity (1). Developing healthy dietary behaviors and creating a good eating environment for children and adolescents is important (2).

The behavior of eating in front of electronic screens was prevalent in children and adolescents (3). In a recent study in Italy, more than 77% of children normally watched TV or played with a tablet/smartphone while eating (4). A study in Chilean showed that 87.5% of the children consumed at least one meal or snack per day while using screens (3). And a study in United Kingdom found more than 70% of the children watched TV during meals (5). In a study among 1,011 Brazilian adolescents, 83.3% reported food consumption while watching TV (6). In addition, a study among pupils in China found that the proportion of eating meals while watching electronic screens and eating snacks while watching electronic screens accounted for 42.0 and 64.2%, respectively (7).

Eating while watching TV was associated with food preferences and food habits (8). A study of Danish children found that frequent consumption of meals during TV viewing, seemed to be associated with generally having unhealthy food preferences and food habits among school-aged children (8). And a study of Portuguese children found that frequently watching TV during meals presented a lower proportion of liking vegetables and a higher proportion of liking sweet dairy products (9). In addition, a study in Brazil found that adolescents that consume food while watching TV had higher weekly consumption of fried foods, sweets, soft drinks and snacks (6).

Eating while watching TV was found associated with children’s overweight and obesity (10–12). In China, a study in 2022 found snacking while watching TV was independently associated with waist circumference among children aged 2–6 years (13). However, this topic was not studied in adolescents in China.

Eating habits established in childhood and adolescence tend to be maintained into adulthood (14). In previous studies, eating while watching TV was found associated with unhealthy food preferences and food habits (8), increased energy intake (15) and obesity (10–12) in children. However, the effects of eating while watching TV on food preferences and overweight/obesity in Chinese adolescents are not clear. The present study used China Health and Nutrition Survey (CHNS) data from 2006 to 2015 to (1) assess the association of eating while watching TV and food preferences in adolescents in China; and (2) explore the influence of eating while watching TV on body mass index (BMI) and overweight/obesity in Chinese adolescents.

## 2 Methods

### 2.1 Study design

The CHNS is an ongoing open cohort, an international collaborative project between the Carolina Population Center at the University of North Carolina at Chapel Hill and the National Institute for Nutrition and Health at the Chinese Center for Disease Control and Prevention (16). It was designed to examine the effects of the health, nutrition and family planning policies and programmes implemented by national and local governments and to see how the social and economic transformation of Chinese society affects the health and nutritional status of its population. The longitudinal CHNS has been conducted since 1989 in eight out of the 23 Chinese provinces (Guangxi, Guizhou, Henan, Hubei, Hunan, Jiangsu, Liaoning, Shandong), and Heilongjiang Province was enrolled as a ninth province in 1997 (17). Three megacities (Beijing, Shanghai, Chongqing) have joined the study since 2011 (18). The 12 participating provinces varied substantially in terms of geography, economic development, public resources, and health indicators, which makes the sample representative (16, 19). A multistage, random cluster process was used to draw the sample in each of these provinces. Counties and cities in each province were stratified by income (low, middle and high), and a weighted sampling scheme was used to randomly select four counties and two cities in each province. Villages and townships within the counties and urban and suburban neighborhoods within the cities were selected randomly. In each community, 20 households were randomly selected, and all household members were interviewed (17).

### 2.2 Participants

**Figure 1:**
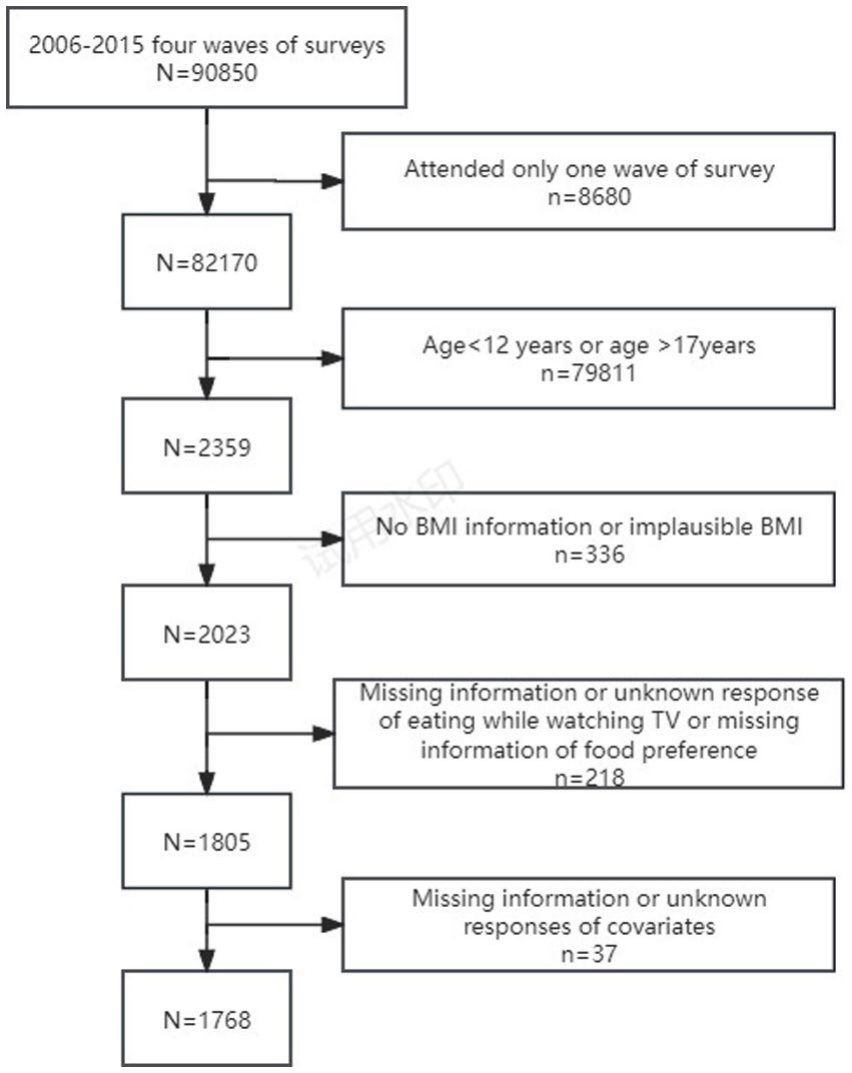
Participant flow chart.

The survey of the frequency of eating meals/snacks while watching TV began in 2004. And individuals above the age of 12 were invited to answer the two questions. As the setting of responses to the questions of eating meals/snacks while watching TV was different between 2004 and after 2004, available CHNS data from surveys conducted in 2006, 2009, 2011 and 2015 was utilized in this study. Hence, 2,359 adolescents aged 12–17 years participated in at least one of the four waves of surveys. Data was collected from participants during face-to-face interviews with trained interviewers at the participants’ homes. We excluded adolescents who had no height and weight information or implausible BMI, those who had missing or unknown response of eating meals/snacks while watching TV, and had missing information of food preferences for fast food, salty snacks, fruits, vegetables and soft drinks. Adolescents with missing information or unknown responses of the following variables were also excluded: age, gender, nationality, education, urbanization, residence and per capital annual family income. The final sample included in the analysis was 1768 adolescents (Figure 1), numbers of adolescents extracted were 480 (2006), 477 (2009), 558 (2011), 253 (2015).

All subjects gave their informed consent for inclusion before they participated in the CHNS, and written informed consent was obtained from a parent or guardian for participants under 16 years old. As the data was public, the data of CHNS is available for all kinds of people.

### 2.3 Measures

#### 2.3.1 Exposure variables: the frequency of eating while watching TV

The frequency of eating snacks/meals while watching TV was assessed by the questions “Do you eat snacks while watching TV?” and “Do you watch TV when you are eating a meal?” Respondents reported the frequency [“very seldom (<1 time/month),” “seldom (1–3 times/month),” “sometimes (1–2 times/week),” “often (3–4 times/week),” “very often (≥5 times/week)” and “unknown”] of the two eating questions. Considering the reality of the question as well as the situation of the data itself, for each of the two questions, responses of “very seldom” and “seldom” were combined as “<1 time/week”; and responses of “sometimes,” “often” and “very often” were combined as “≥1 time/week” (20).

All field workers have been trained nutritionists who are otherwise professionally engaged in nutrition work in their own counties and who have participated in other national surveys (21). Almost all interviewers have been graduates of post-secondary schools; many have had four-year degrees (21).

#### 2.3.2 Outcome variables: food preferences, BMI and overweight/obesity

Respondents were asked to describe how much they like the five kinds of food (“dislike very much,” “dislike,” “neutral,” “like,” “like very much” or “does not eat this food”): (1) fast food (fried chicken, pizza, hamburgers, etc.); (2) salty snack foods (potato chips, pretzels, French fries, etc.); (3) fruits; (4) vegetables, and (5) soft drinks and sugary fruit drinks. According to previous studies (22, 23), the responses to each question for food preferences were collapsed into two categories (“like” and “dislike”). Specifically, responses of both “like very much” and “like” were grouped into one category of “like,” whereas responses of “dislike very much,” “dislike,” “neutral” or “does not eat this food” were grouped into the other category of “dislike.”

The height and weight of children were measured by at least two trained health workers who followed standard protocol and techniques, with one worker taking the measurements while a second health worker recording the readings (24). Bodyweight was measured in light indoor clothing without shoes to the nearest tenth of a kilogram with a beam balance scale; height was measured without shoes to the nearest tenth of a centimeter, using a portable stadiometer (24). BMI, defined as the body weight in kilograms divided by the squared body height in meters, is used here as the indicator of adolescents’ overweight and obesity. The International Obesity Task Force cut-off of body mass index was used for defining overweight/obesity among children (25).

#### 2.3.3 Covariates

Age, gender, ethnicity, education, residence, urbanization index and *per capita* annual family income of the children were included in this study. Self-reported education as indicated in the questionnaire was allocated to one of three categories (illiterate/primary school, junior middle school, high middle school or higher) (26). The urbanization index was recoded into tertiles (low, medium, and high) (26). *Per capita* annual family income was recoded into tertiles (low, medium, and high) (26).

### 2.4 Data analyses

Descriptive statistics were used for the sample characteristics. The categorical variables were described using frequency and percentage, and the continuous variables were described using mean and standard deviation (SD). The chi-square test was used to compare differences between adolescents with overweight/obesity and without overweight/obesity. To determine if the relationships between eating while watching TV and food preferences and BMI and overweight/obesity among adolescents existed, odds ratios (ORs)/beta coefficient (β) and 95% confidence intervals (CIs) for the outcome variables were calculated using mixed effect models adjusting for covariates. All statistical tests were conducted using STATA software (Version 15, StataCorp, College Station, TX, USA). Statistical significance was considered when *p* < 0.05 (two-sided).

## 3 Results

### 3.1 Sample characteristics

The characteristics of the adolescents were shown in Table 1. Our study included 1768 adolescents, of whom 60.1% aged 12–14 years, 52.9% were boys, 65.6% resided in rural areas, and 63.8% were in junior middle school. Among the adolescents, 45.4% of them eat snacks while watching TV ≥1 time/week and 30.7% eat meals while watching TV ≥1 time/week. In addition, adolescents aged 12–14 had a higher proportion of overweight/obesity than those aged 15–17 (*p* < 0.05). Boys had a higher proportion of overweight/obesity than girls (*p* < 0.001), and Han nationality had a higher proportion of being overweight/obesity than minority (*p* < 0.05). The proportion of adolescents’ overweight/obesity was significantly higher when they had a high per capital annual family income (*p* < 0.001).

**Table 1:** Sample characteristics of participants (*n* = 1768).

Characteristics	Total populations	Overweight/obesity		*p*-value
		No (*n* = 1,560)	Yes (*n* = 208)	
Age group (*n*, %)				
12–14	1,063 (60.1)	924 (59.2)	139 (66.8)	**0.036**
15–17	705 (39.9)	636 (40.8)	69 (33.2)	
Gender (*n*, %)				
Boy	935 (52.9)	800 (51.3)	135 (64.9)	**<0.001**
Girl	833 (47.1)	760 (48.7)	73 (35.1)	
Nationality (*n*, %)				
Han	1,510 (85.4)	1,320 (84.6)	190 (91.3)	**0.010**
Minority	258 (14.6)	240 (15.4)	18 (8.7)	
Education (*n*, %)				
Illiterate/primary school	272 (15.4)	237 (15.2)	35 (16.8)	0.58
Junior middle school	1,128 (63.8)	993 (63.7)	135 (64.9)	
High middle school or higher	368 (20.8)	330 (21.2)	38 (18.3)	
Urbanization (*n*, %)				
Low	590 (33.4)	532 (34.1)	58 (27.9)	0.11
Medium	589 (33.3)	520 (33.3)	69 (33.2)	
High	589 (33.3)	508 (32.6)	81 (38.9)	
Residence (*n*, %)				
Urban	608 (34.4)	526 (33.7)	82 (39.4)	0.10
Rural	1,160 (65.6)	1,034 (66.3)	126 (60.6)	
Per capital annual family income (*n*, %)				
Low	590 (33.4)	552 (35.4)	38 (18.3)	**<0.001**
Medium	589 (33.3)	517 (33.1)	72 (34.6)	
High	589 (33.3)	491 (31.5)	98 (47.1)	
Survey year (*n*, %)				
2006	480 (27.1)	448 (28.7)	32 (15.4)	**<0.001**
2009	477 (27.0)	425 (27.2)	52 (25.0)	
2011	558 (31.6)	484 (31.0)	74 (35.6)	
2015	253 (14.3)	203 (13.0)	50 (24.0)	
Eat snacks while watching TV (*n*, %)				
<1 time/week	966 (54.6)	847 (54.3)	119 (57.2)	0.43
≥1 time/week	802 (45.4)	713 (45.7)	89 (42.8)	
Eat meals while watching TV (*n*, %)				
<1 time/week	1,225 (69.3)	1,089 (69.8)	136 (65.4)	0.19
≥1 time/week	543 (30.7)	471 (30.2)	72 (34.6)	
Preference for fast food (*n*, %)				
Dislike	949 (53.7)	841 (53.9)	108 (51.9)	0.59
Like	819 (46.3)	719 (46.1)	100 (48.1)	
Preference for salty snacks (*n*, %)				
Dislike	792 (44.8)	700 (44.9)	92 (44.2)	0.86
Like	976 (55.2)	860 (55.1)	116 (55.8)	
Preference for fruits (*n*, %)				
Dislike	253 (14.3)	217 (13.9)	36 (17.3)	0.19
Like	1,515 (85.7)	1,343 (86.1)	172 (82.7)	
Preference for vegetables (*n*, %)				
Dislike	543 (30.7)	473 (30.3)	70 (33.7)	0.33
Like	1,225 (69.3)	1,087 (69.7)	138 (66.3)	
Preference for soft drinks (*n*, %)				
Dislike	603 (34.1)	534 (34.2)	69 (33.2)	0.76
Like	1,165 (65.9)	1,026 (65.8)	139 (66.8)	

Results in bold are statistically significant (*p* < 0.05).

### 3.2 Association between eating while watching TV and food preferences among the adolescents

The results showed that when adjusting for confounding factors, adolescents eating meals ≥1 time/week while watching TV were more likely to prefer fast food (OR 2.18, 95% CI 1.71–2.78), salty snacks (OR 2.40, 95% CI 1.83–3.14) and soft drinks (OR 1.91, 95% CI 1.46–2.50), while less likely to prefer vegetables (OR 0.72, 95% CI 0.57–0.92) than those eating meals <1 time/week. In addition, compared with adolescents eating snacks <1 time/week while watching TV, adolescents eating snacks ≥1 time/week while watching TV were also more likely to prefer fast food (OR 1.38, 95% CI 1.10–1.72), salty snacks (OR 1.57, 95% CI 1.25–1.98) and soft drinks (OR 1.48, 95% CI 1.16–1.88) (Table 2).

**Table 2:** Association (OR, 95% CI) between eating while watching TV and food preferences among adolescents attending China Health and Nutrition Survey (2006–2015) (*n* = 1768).

Food preference	Eat meals while watching TV (time/week)^a^		Eat snacks while watching TV (time/week)^a^	
	<1	≥1	<1	≥1
Fast food	Reference	2.18 (1.71–2.78)**	Reference	1.38 (1.10–1.72)**
Salty snacks	Reference	2.40 (1.83–3.14)**	Reference	1.57 (1.25–1.98)**
Fruits	Reference	1.03 (0.77–1.39)	Reference	1.02 (0.78–1.35)
Vegetables	Reference	0.72 (0.57–0.92)**	Reference	0.88 (0.70–1.10)
Soft drinks	Reference	1.91 (1.46–2.50)**	Reference	1.48 (1.16–1.88)**

a Adjusted for age group, gender, nationality, education, urbanization, residence and per capital annual family income. *p < 0.05, **p < 0.01. Results in bold are statistically significant (*p* < 0.05).

### 3.3 Mixed effect models on the associations between eating while watching TV and overweight/obesity of adolescents

Logistic regression analyses (Table 3) showed that adolescents eating snacks while watching TV ≥1 time/week were more likely to be overweight and obesity than those eating snacks while watching TV <1 time/week when adjusting the confounding factors (OR = 7.16; 95% CI 1.39–36.93). However, eating meals while watching TV ≥1time/week was not found associated with adolescents’ BMI or overweight/obesity.

**Table 3:** Association (OR, 95%CI) between eating while watching TV and overweight and obesity among adolescents attending the China Health and Nutrition Survey (2006–2015) (*n* = 1768).

	Adjusted model^a^ (BMI as categorical variable)	Adjusted model^a^ (BMI as continuous variable)
Independent variables	OR (95% CI)	β (95% CI)
Eat meals while watching TV (time/week)		
<1	Reference	Reference
≥1	2.34 (0.63–8.65)	0.04 (−0.25–0.33)
Eat snacks while watching TV (time/week)		
<1	Reference	Reference
≥1	7.16 (1.39–36.93)*	−0.10 (−0.36–0.16)

a Adjusted for age group, gender, nationality, education, urbanization, residence, per capital annual family income and food preferences. *p < 0.05, **p < 0.01. Results in bold are statistically significant (*p* < 0.05).

## 4 Discussion

Among the adolescents, 30.7% eat meals while watching TV ≥1 time/week and 45.4% eat snacks while watching TV ≥1 time/week. The proportion of liking fast food, salty snacks and soft drinks accounted for 46.3, 55.2, and 65.9%, respectively. This reflected that preference for unhealthy food and eating while watching TV were prevalent in Chinese adolescents. The study also found that eating while watching TV ≥1 time/week was positively associated with unhealthy food preferences and negatively associated with healthy food preferences. In addition, adolescents eating snacks while watching TV ≥1 time/week were more likely to be overweight and obesity.

In this study, adolescents eating while watching TV ≥1 time/week were more likely to prefer fast food, salty snacks and soft drinks than those eating while watching TV <1 time/week. In addition, the study demonstrated that eating meals while watching TV ≥1 time/week was negatively associated with the preference of vegetables. These results were similar to previous studies (6, 8, 9). The possible reasons were as follows. Firstly, watching TV during meals resulted in lower attention to the sensory characteristics of food, leading to lower preferences for less flavorous foods, with bitter and sour tastes and higher preferences for tasty sweet foods (9). Secondly, as familiarity with one food was an important determinant for the preference for that food (27), exposure of television (TV) food advertisements could increase adolescents’ familiarity and preference with advertised food or branded food items (28). However, non-core foods (high in undesirable nutrients or energy, as defined by dietary standards) comprised 53 to 87% among the TV food advertisements (29). Given the primacy of children’s likes and dislikes, the assessment of food preferences can be especially useful as predictors of food consumption patterns (30).

This study found that adolescents eating snacks while watching TV ≥1time/week were more likely to be overweight and obesity than those eating snacks while watching TV <1 time/week, which was similar to previous studies (31–34). The possible reasons are as follows. Firstly, previous studies have found that portion sizes of biscuits, chocolate, crisps and savoury snacks were larger when eaten in front of the TV among children (35). Large portion sizes of palatable, energy dense foods produce a reliable increase in energy intake compared to small or regular portion sizes (36). A study in Chilean also found that large percentage of daily energy is consumed while using screens among children and adolescents (3). Secondly, exposure of TV food advertisements can increase children’s preference for branded food items (28), which often contains a lot of non-core foods. Thirdly, eating while watching TV tends to distract children’s attention to food intake and disrupt the ability to adequately respond to normal internal satiety cues, which can easily lead to mindless eating, eating too fast, then make children eat more food (37). Fourthly, the advertisements of fast food and unhealthy snacks may have a negative impact on children’s food preferences (38) and food choices (39), making it easy for children to choose high-calorie and nutrients-poor food (40). Fifthly, increased sedentary time induced by watching electronic screens may lead to reduced physical activity and energy expenditure, which can increase the risk of overweight and obesity in children (41). In addition, screen exposure is also associated with sleep deprivation, which is in turn associated with overweight/obesity (42).

The study did not find the association between eating meals while watching TV and adolescents’ BMI or overweight/obesity, which was different with previous studies (33). A previous study found that there was a negative relationship between never watching TV at lunch and dinner and overweight or obesity among children in eight European countries (33). And a systematic review in 2018 found a positive association between eating dinner while TV viewing and overweight existed in children (34). It is worth noting that, in the present study, the association between eating meals while watching TV ≥1 time/week and fast food, salty snacks and soft drinks preference is stronger than between eating snacks while watching TV ≥1 time/week and fast food, salty snacks and soft drinks preference. However, eating meals while watching TV was not associated with overweight/obesity, but eating snacks while watching TV was. One possible reason may be that food preferences and actual food intake is not necessarily highly correlate, which may be caused by parents’ restrictions (43, 44). Therefore, adolescents with fast food, salty snacks and soft drinks preference may have not very much these food intake. Another possible reason may be related to that the proportion of TV viewing during snacks ≥1 time/week (45.4%) was higher than the proportion of TV viewing during meals ≥1 time/week (30.7%) in the study. As eating while watching TV was found associated with unhealthy food preferences and food habits (8), increased energy intake (15), and obesity (10–12, 34) in children and adolescents. Adolescents eating snacks while watching TV ≥1 time/week may have more chances to take unhealthy food and higher risk of being obesity than those eating meals while watching TV ≥1 time/week.

In the study, we pooled together data from surveys conducted in 2006, 2009, 2011 and 2015. However, it’s quite clear that both food and TV show schedules have deeply changed over the last 20 years. Therefore, we have separately analyzed the association of eating while watching TV and food preference and overweight/obesity in 2006, 2009, 2011 and 2015 in Supplementary Tables. The survey year stratified analysis showed that the association of eating while watching TV and food preference in 2006, 2009, 2011 and 2015 was similar to the results of the present study. However, no significant association was found between eating while watching TV and overweight and obesity in 2006, 2009, 2011 and 2015, respectively. This is different from the present study, which may be due to that the sample size included in each survey year is relatively small, which was 529 (2006), 483 (2009), 619 (2011), 302 (2015).

Given that daily screen exposure (TV, but also smartphones, computer, videogames, etc.) seems to be an unavoidable part of everyday life, developing specific mobile apps may become an important channel for health-promoting interventions. In addition, community-based participatory research (CBPR) is a collaborative, partnership approach to research that directly and equitably involves community members in all phases of the research process (45). CBPR have been carried out in several populations, including children (45). It not only considers the multifactorial influences impacting child health but also allow children to have a voice about their own health and illness (45, 46). Brilliant strategies may lie in implementing web-based CBPR and integrated knowledge translation approaches (45, 46). Examples of web-based CBPR have been shown to successfully involve the knowledge users (47, 48). Therefore, implementing web-based CBPR to reduce eating while watching TV may be a practical strategy, such as developing specific mobile apps, creating interactive websites, creating WeChat groups (49), and so on. This strategy was suggested to be pursued in children, adolescents and their caregivers. In addition, the results of the study would reflect that Chinese government should formulate child nutrition related policy about reducing eating while watching TV or developing healthy dietary behaviors while watching TV. And health educators should conduct food and nutrition education about not eating while watching TV among adolescents and their caregivers to promote healthy food preferences and prevent overweight and obesity of adolescents.

To the best of our knowledge, this was the first study to explore the influence of eating while watching TV on food preferences, BMI and overweight/obesity among adolescents in China. The findings may have some practical implications for healthy food preferences promotion and obesity prevention among adolescents by proposing not eating while watching TV or eating moderate healthy foods while watching TV.

However, several limitations should be proposed. Firstly, the assessment of food preferences was made through questionnaires rather than by tasting the foods, and factors such as the usual method of consumption (type of cooking, food combinations, fat content, among others) were not controlled, which may affect the accuracy of the calculation of food preferences. Secondly, as both food and TV show schedules have deeply changed over the last 20 years, which may have some influence on the results. Therefore, the kind, composition and quantity of food consumed while watching TV, and the content and duration of TV viewing need to be considered in future studies. We hope these information can be collected in future study to further explore the mechanism of the association between eating while watching TV and food preferences and obesity in adolescents.

## 5 Conclusion

This study showed that adolescents eating while watching TV ≥1 time/week was positively associated with the preference for unhealthy foods than those eating while watching TV <1 time/week. Adolescents eating snacks while watching TV ≥1 time/week were more likely to be overweight and obesity. The study demonstrated that avoiding eating while watching TV may be meaningful to promote healthy preference and prevent overweight/obesity in Chinese adolescents, which may need the joint efforts of family, school and society.

## Data Availability

The raw data supporting the conclusions of this article will be made available by the authors, without undue reservation.
