# Supplementary material for: Influence of eating while watching TV on food preference and overweight/obesity among adolescents in China: a longitudinal study
Source: Front Public Health. 2024 Sep 17;12:1423383. doi: 10.3389/fpubh.2024.1423383 (PMC11442226; doi:10.3389/fpubh.2024.1423383)
Supplement: Supplementary file 1 [file Table_1.docx]

**Supplement table 1** Association (OR, 95% CI) between eating while watching TV and food preference among adolescents attending the 2006 China Health and Nutrition Survey (n = 529)

| **Food preference** | **Eat meals while watching TV (time/week) ^a^** | | **Eat snacks while watching TV (time/week) ^a^** | |
| --- | --- | --- | --- | --- |
|  | **<1** | **≥1** | **<1** | **≥1** |
| **Fast food** | Reference | 1.66 (1.11-2.47)* | Reference | 0.98 (0.68-1.42) |
| **Salty snacks** | Reference | 1.19 (0.81-1.75) | Reference | 1.00 (0.70-1.42) |
| **Fruits** | Reference | 1.10 (0.60-2.00) | Reference | 1.18 (0.69-2.01) |
| **Vegetables** | Reference | 0.72 (0.47-1.09) | Reference | 0.65 (0.44-0.96)* |
| **Soft drinks** | Reference | 1.14 (0.77-1.68) | Reference | 1.26 (0.88-1.81) |

^a^ Adjusted for agegroup, gender, nationality, education, urbanization, residence and per capital annual family income.

* *p* < 0.05, ** *p* < 0.01.

**Supplement table 2** Association (OR, 95%CI) between eating while watching TV and overweight and obesity among adolescents attending the 2006 China Health and Nutrition Survey (n = 529)

| **Independent variables** | **Adjusted model ^a^ (BMI as categorical variable)** |  | **Adjusted model ^a^ (BMI as continuous variable)** |
| --- | --- | --- | --- |
|  | **OR (95% CI)** |  | **β (95% CI)** |
| **Eat meals while watching TV (time/week)** |  |  |  |
| <1 | Reference |  | Reference |
| ≥1 | 1.39 (0.66–2.93) |  | 0.43 (-0.07–0.93) |
| **Eat snacks while watching TV(time/week)** |  |  |  |
| <1 | Reference |  | Reference |
| ≥1 | 0.87 (0.43–1.77) |  | 0.04 (-0.41–0.50) |

^a^ Adjusted for agegroup, gender, nationality, education, urbanization, residence, per capital annual family income and food preference.

* *p* < 0.05, ** *p* < 0.01.

**Supplement table 3** Association (OR, 95% CI) between eating while watching TV and food preference among adolescents attending the 2009 China Health and Nutrition Survey (n = 483)

| **Food preference** | **Eat meals while watching TV (time/week) ^a^** | | **Eat snacks while watching TV (time/week) ^a^** | |
| --- | --- | --- | --- | --- |
|  | **<1** | **≥1** | **<1** | **≥1** |
| **Fast food** | Reference | 2.55 (1.67-3.89)** | Reference | 1.44 (0.99-2.11) |
| **Salty snacks** | Reference | 2.14 (1.40-3.27)** | Reference | 1.80 (1.23-2.63)** |
| **Fruits** | Reference | 1.12 (0.62-2.03) | Reference | 0.60 (0.35-1.02) |
| **Vegetables** | Reference | 0.64 (0.41-1.00)* | Reference | 0.77 (0.50-1.17) |
| **Soft drinks** | Reference | 1.47 (0.94-2.32) | Reference | 1.02 (0.68-1.53) |

^a^ Adjusted for agegroup, gender, nationality, education, urbanization, residence and per capital annual family income.

* *p* < 0.05, ** *p* < 0.01.

**Supplement table 4** Association (OR, 95%CI) between eating while watching TV and overweight and obesity among adolescents attending the 2009 China Health and Nutrition Survey (n = 483)

| **Independent variables** | **Adjusted model ^a^ (BMI as categorical variable)** |  | **Adjusted model ^a^ (BMI as continuous variable)** |
| --- | --- | --- | --- |
|  | **OR (95% CI)** |  | **β (95% CI)** |
| **Eat meals while watching TV (time/week)** |  |  |  |
| <1 | Reference |  | Reference |
| ≥1 | 1.76 (0.88–3.53) |  | 0.23 (-0.37–0.83) |
| **Eat snacks while watching TV(time/week)** |  |  |  |
| <1 | Reference |  | Reference |
| ≥1 | 1.19 (0.64–2.22) |  | 0.20 (-0.34–0.75) |

^a^ Adjusted for agegroup, gender, nationality, education, urbanization, residence, per capital annual family income and food preference.

* *p* < 0.05, ** *p* < 0.01.

**Supplement table 5** Association (OR, 95% CI) between eating while watching TV and food preference among adolescents attending the 2011 China Health and Nutrition Survey (n = 619)

| **Food preference** | **Eat meals while watching TV (time/week) ^a^** | | **Eat snacks while watching TV (time/week) ^a^** | |
| --- | --- | --- | --- | --- |
|  | **<1** | **≥1** | **<1** | **≥1** |
| **Fast food** | Reference | 1.71 (1.19-2.45)** | Reference | 1.66 (1.19-2.33)** |
| **Salty snacks** | Reference | 2.75 (1.89-4.02)** | Reference | 1.67 (1.20-2.32)** |
| **Fruits** | Reference | 0.67 (0.42-1.06) | Reference | 1.11 (0.71-1.73) |
| **Vegetables** | Reference | 0.65 (0.45-0.94)* | Reference | 0.94 (0.67-1.33) |
| **Soft drinks** | Reference | 2.58 (1.70-3.90)** | Reference | 1.71 (1.20-2.43)** |

^a^ Adjusted for agegroup, gender, nationality, education, urbanization, residence and per capital annual family income.

* *p* < 0.05, ** *p* < 0.01.

**Supplement table 6** Association (OR, 95%CI) between eating while watching TV and overweight and obesity among adolescents attending the 2011 China Health and Nutrition Survey (n = 619)

| **Independent variables** | **Adjusted model ^a^ (BMI as categorical variable)** |  | **Adjusted model ^a^ (BMI as continuous variable)** |
| --- | --- | --- | --- |
|  | **OR (95% CI)** |  | **β (95% CI)** |
| **Eat meals while watching TV (time/week)** |  |  |  |
| <1 | Reference |  | Reference |
| ≥1 | 1.59 (0.93–2.71) |  | 0.42 (-0.16–1.00) |
| **Eat snacks while watching TV(time/week)** |  |  |  |
| <1 | Reference |  | Reference |
| ≥1 | 1.14 (0.70–1.87) |  | 0.14 (-0.38–0.66) |

^a^ Adjusted for agegroup, gender, nationality, education, urbanization, residence, per capital annual family income and food preference.

* *p* < 0.05, ** *p* < 0.01.

**Supplement table 7** Association (OR, 95% CI) between eating while watching TV and food preference among adolescents attending the 2015 China Health and Nutrition Survey (n = 302)

| **Food preference** | **Eat meals while watching TV (time/week) ^a^** | | **Eat snacks while watching TV (time/week) ^a^** | |
| --- | --- | --- | --- | --- |
|  | **<1** | **≥1** | **<1** | **≥1** |
| **Fast food** | Reference | 2.38 (1.44-3.93)** | Reference | 1.51 (0.93-2.44) |
| **Salty snacks** | Reference | 3.01 (1.81-5.00)** | Reference | 2.51 (1.54-4.11)** |
| **Fruits** | Reference | 1.20 (0.65-2.20) | Reference | 0.97 (0.54-1.74) |
| **Vegetables** | Reference | 1.04 (0.64-1.71) | Reference | 0.94 (0.58-1.52) |
| **Soft drinks** | Reference | 2.63 (1.53-4.53)** | Reference | 2.73 (1.61-4.61)** |

^a^ Adjusted for agegroup, gender, nationality, education, urbanization, residence and per capital annual family income.

* *p* < 0.05, ** *p* < 0.01.

**Supplement table 8** Association (OR, 95%CI) between eating while watching TV and overweight and obesity among adolescents attending the 2015 China Health and Nutrition Survey (n = 302)

| **Independent variables** | **Adjusted model ^a^ (BMI as categorical variable)** |  | **Adjusted model ^a^ (BMI as continuous variable)** |
| --- | --- | --- | --- |
|  | **OR (95% CI)** |  | **β (95% CI)** |
| **Eat meals while watching TV (time/week)** |  |  |  |
| <1 | Reference |  | Reference |
| ≥1 | 0.85 (0.45–1.61) |  | -0.65 (-1.77–0.47) |
| **Eat snacks while watching TV(time/week)** |  |  |  |
| <1 | Reference |  | Reference |
| ≥1 | 0.63 (0.34–1.19) |  | -0.92 (-2.02–0.18) |

^a^ Adjusted for agegroup, gender, nationality, education, urbanization, residence, per capital annual family income and food preference.

* *p* < 0.05, ** *p* < 0.01.
